# 1,4-Dihydroxy­quinoxaline-2,3(1*H*,4*H*)-dione

**DOI:** 10.1107/S1600536808003784

**Published:** 2008-02-08

**Authors:** Rajab Abu-El-Halawah, Basem Fares Ali, Mohammad M. Ibrahim, Jalal A. Zahra, Wolfgang Frey

**Affiliations:** a Department of Chemistry, Al al-Bayt University, Mafraq, Jordan; b Institut für Organische Chemie der Universität Stuttgart, Pfaffenwaldring 55, 70569 Stuttgart, Germany; c Chemistry Department, University of Jordan, Amman, Jordan

## Abstract

The asymmetric unit of the title compound, C_8_H_6_N_2_O_4_, contains one half-mol­ecule; a twofold rotation axis bisects the molecule. The quinoxaline ring is planar, which can be attributed to electron delocalization. In the crystal structure, inter­molecular O—H⋯O hydrogen bonds link the mol­ecules into *R*_2_^2^(10) motifs, leading to layers, which inter­act *via* phen­yl–phenyl inter­actions (C⋯C distances in the range 3.238–3.521 Å).

## Related literature

For general background, see: Zarranz *et al.* (2004 ▶); Chowdhury *et al.* (2004 ▶); Monge *et al.* (1995 ▶); Fuchs *et al.* (2001 ▶); Dance (1996 ▶); Bernstein *et al.* (1995 ▶). For related literature, see: Elina & Tsyrul’nikova (1963 ▶); Akkurt *et al.* (2004 ▶); Mustaphi *et al.* (2001 ▶); Oxtoby *et al.* (2005 ▶); Ley & Seng (1975 ▶); For bond-length data, see: Allen *et al.* (1987 ▶);
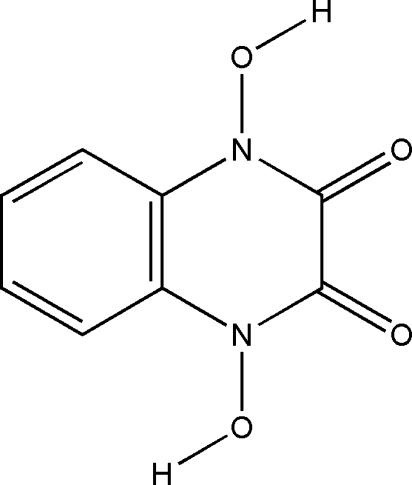


## Experimental

### 

#### Crystal data

C_8_H_6_N_2_O_4_
*M* *_r_* = 194.15
Orthorhombic, 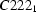

*a* = 4.2562 (6) Å
*b* = 17.630 (3) Å
*c* = 10.4775 (17) Å
*V* = 786.2 (2) Å^3^
*Z* = 4
Mo *K*α radiation
μ = 0.14 mm^−1^
*T* = 294 (2) K
0.50 × 0.20 × 0.10 mm

#### Data collection

Nicolet P3 diffractometer
Absorption correction: none
1004 measured reflections
529 independent reflections
437 reflections with *I* > 2σ(*I*)
*R*_int_ = 0.022
3 standard reflections every 50 reflections intensity decay: 2%

#### Refinement

*R*[*F* ^2^ > 2σ(*F* ^2^)] = 0.034
*wR*(*F* ^2^) = 0.071
*S* = 1.07
529 reflections
69 parameters
H atoms treated by a mixture of independent and constrained refinement
Δρ_max_ = 0.12 e Å^−3^
Δρ_min_ = −0.15 e Å^−3^

### 

Data collection: *P3/PC Data Collection Software* (Siemens, 1991 ▶); cell refinement: *P3/PC Data Collection Software*; data reduction: *SHELXTL-Plus* (Sheldrick, 2008 ▶); program(s) used to solve structure: *SHELXS97* (Sheldrick, 2008 ▶); program(s) used to refine structure: *SHELXL97* (Sheldrick, 2008 ▶); molecular graphics: *SHELXTL-Plus*; software used to prepare material for publication: *SHELXL97* and *PLATON* (Spek, 2003 ▶).

## Supplementary Material

Crystal structure: contains datablocks I, global. DOI: 10.1107/S1600536808003784/hk2425sup1.cif

Structure factors: contains datablocks I. DOI: 10.1107/S1600536808003784/hk2425Isup2.hkl

Additional supplementary materials:  crystallographic information; 3D view; checkCIF report

## Figures and Tables

**Table 1:** Hydrogen-bond geometry (Å, °)

*D*—H⋯*A*	*D*—H	H⋯*A*	*D*⋯*A*	*D*—H⋯*A*
O1—H1⋯O2^i^	0.96 (3)	1.63 (3)	2.584 (2)	174 (3)

Symmetry code: (i) 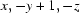.

**Fig. 1.**
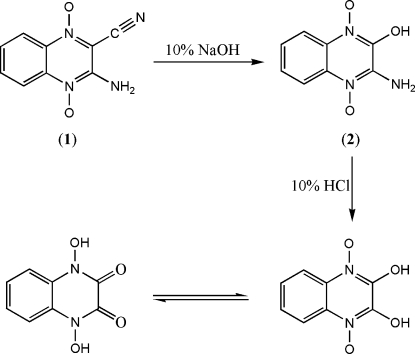
Schematic representation for the steps through which reaction proceeds.

**Fig. 2.**
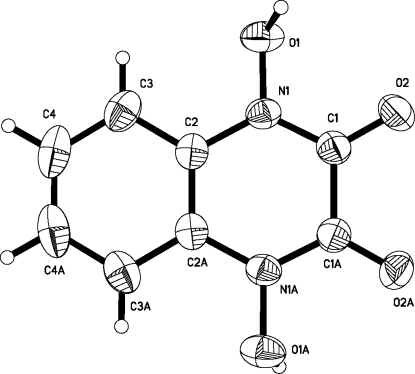
The molecular structure of the title molecule, with the atom-numbering scheme. Displacement ellipsoids are drawn at the 50% probability level.

**Fig. 3.**
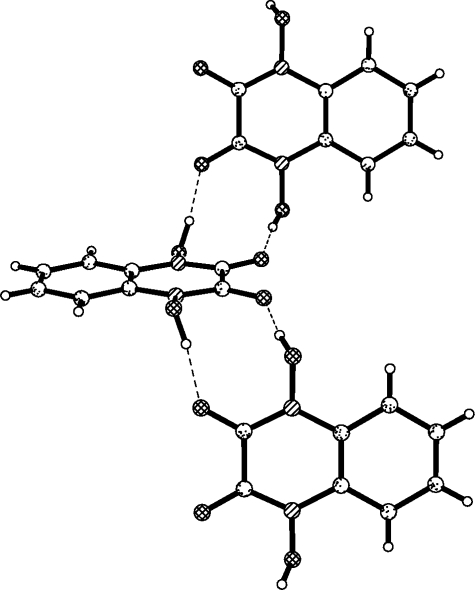
Part of the crystal structure of (I), showing the formation of R22(10) motifs.

**Fig. 4.**
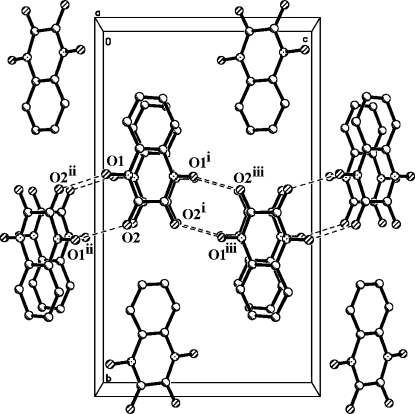
A packing diagram of (I), showing the layers of molecules parallel to c axis. All hydrogen atoms were omitted for clarity. Hydrogen bonds are shown as dashed lines [symmetry codes: (i) -x, y, -z + 1/2, (ii) x, -y + 1, -z].

## supplementary crystallographic information

### Comment

Quinoxalines are of interest owing to their biological activities. They seem to have very interesting anticancer activity (Zarranz *et al.*, 2004). For example, 3-aminoquinoxaline-2-carbonitrile 1,4-dioxide have been studied extensively as bioreductive cytotoxic agent. It was found to be an efficient agent and causes redox-activated DNA damage (Chowdhury *et al.*, 2004), even more active than the first drug clinically used as bioreductive cytotoxic agent (Monge *et al.*, 1995; Fuchs *et al.*, 2001). A nonconvenient synthesis of the title compound, (I), was reported previously (Elina & Tsyruľ nikova, 1963), *via* the hydrolysis of 2-amino-3-hydroxyquinoxaline 1,4-dioxide, which results as a side product (4%) from oxidation of 2-acetamidoquinoxaline with acetic peroxide acid using boiling HCl solution. We report herein a novel simple synthetic method for (I), along with its crystal structure.

The new synthetic strategy for (I), (Fig. 1), is based on the reaction of (1) with NaOH solution, yielding (2) in an S_N_Ar reaction, which upon hydrolysis with boiling HCl solution, *via* protonation of amine followed by the attack of water molecule, yielded (I) in a good amount (90%).

The asymmetric unit of the title compound, (I), (Fig. 2) contains one half-molecule. The quinoxaline ring is planar, which can be attributed to a wide range of electron delocalization. Bond lengths and angles are in accordance with the corresponding reported values in 1,4-dihydroquinoxaline -2,3-dione core (Oxtoby *et al.*, 2005) and other similar *N*-alkyl quinoxalines (Akkurt *et al.*, 2004; Mustaphi *et al.*, 2001). The existence of (I) in the dione form is evident from C1—O2 [1.226 (3) Å] bond, being smaller than a pure single bond, which confirms the double bond character (Allen *et al.*, 1987). The C1—C1^i^ [1.503 (4) Å] bond has single bond character compared to multiple bond characters in C2—C2^i^ [1.381 (4) Å], C2—C3 [1.391 (3) Å], C3—C4 [1.382 (3) Å] and C4—C4^i^ [1.372 (6) Å] [symmetry code: (i) -*x*, *y*, 1/2 - *z*]. The N1—C1 [1.345 (3) Å] bond is significantly shorter than N1—C2 [1.404 (3) Å] and it is an intermediate between those typical for the corresponding single and double bonds, suggesting some degree of delocalization. The N1—C1 bond length is closer to the average C*_ar_*—N*_sp_^2 ^*(planar) value of 1.353 (7) Å rather than the C*_ar_*—N*_sp_^3^* (pyramidal) value of 1.419 (17) Å (Allen *et al.*, 1987), with the sum of the bond angles around atom N1 [359.81 (18)°], indicating *sp^2^* hybridization.

In the crystal structure, intermolecular O—H···O hydrogen bonds (Table 1) link the molecules into *R*_2_^2^(10) motifs (Fig. 3) (Bernstein *et al.*, 1995) leading to layers running along the *c* axis (Fig. 4). Molecules within layers are further interacting *via* phenyl···phenyl interactions (Dance, 1996), where the layers parallel to *a* axis interact in an offset stacking motif (C···C distances in the range of 3.238–3.521 Å).

### Experimental

For the preparation of (I), to a suspension solution of (1) (2.02 g, 10 mmol) (Ley & Seng, 1975) in ethanol (20 ml), NaOH (20 ml, 10%) was added to give a deep blue solution. After refluxing for 5 h, the brown solution was allowed to cool to room temperature. The resulting mixture was then treated with HCl (30 ml, 10%), refluxed for another 5 h and then allowed to stand undisturbed. The resulting residual brown solid was filtered off, washed with cold water (5 ml) and then by cold ethanol (5 ml). The title compound, (I), was recrystallized from ethanol solution (yield; 1.76 g, 90%, m.p. 535–536 K decomposition). Analysis found: C 49.45, H 3.27, N 14.41%; C_8_H_6_N_2_O_4_ requires: C 49.49, H 3.12, N 14.43%. ^1^H NMR (300 MHz, *DMSO-d_6_*): δ = 7.36 (m, 2H; H4/H7), 7.56 (m, 2H; H5/H6); ^13^C NMR (75 MHz, *DMSO-d_6_*): δ = 111.6 (C4/C7), 123.3 (C5/C6), 124.0 (C4a/C7a), 150.4 (C2/C3) p.p.m.. ESI: m/*z* = 217.02 (C_8_H_6_N_2_O_4_Na).

### Refinement

H atom (for OH) was located in a difference synthesis and refined isotropically [O—H = 0.96 (3) Å; *U*_iso_(H) = 0.072 (10) Å^2^]. The remaining H atoms were positioned geometrically, with C—H = 0.93 Å for aromatic H and constrained to ride on their parent atoms, with *U*_iso_(H) = 1.2*U*_eq_(C).

### Crystal data

**Table:**

C_8_H_6_N_2_O_4_	*F*_000_ = 400
*M**_r_* = 194.15	*D*_x_ = 1.640 Mg m^−^^3^
Orthorhombic, *C*222_1_	Mo *K*α radiation λ = 0.71073 Å
Hall symbol: C 2c 2	Cell parameters from 20 reflections
*a* = 4.2562 (6) Å	θ = 14–16º
*b* = 17.630 (3) Å	µ = 0.14 mm^−^^1^
*c* = 10.4775 (17) Å	*T* = 294 (2) K
*V* = 786.2 (2) Å^3^	Plates, colourless
*Z* = 4	0.50 × 0.20 × 0.10 mm

### Data collection

**Table:**

Nicolet P3 diffractometer	*R*_int_ = 0.022
Radiation source: fine-focus sealed tube	θ_max_ = 27.0º
Monochromator: graphite	θ_min_ = 2.3º
*T* = 294(2) K	*h* = 0→5
Wyckoff scan	*k* = 0→22
Absorption correction: none	*l* = −13→13
1004 measured reflections	3 standard reflections
529 independent reflections	every 50 reflections
437 reflections with *I* > 2σ(*I*)	intensity decay: 2%

### Refinement

**Table:**

Refinement on *F*^2^	Hydrogen site location: inferred from neighbouring sites
Least-squares matrix: full	H atoms treated by a mixture of independent and constrained refinement
*R*[*F*^2^ > 2σ(*F*^2^)] = 0.034	*w* = 1/[σ^2^(*F*_o_^2^) + (0.0208*P*)^2^ + 0.4073*P*] where *P* = (*F*_o_^2^ + 2*F*_c_^2^)/3
*wR*(*F*^2^) = 0.071	(Δ/σ)_max_ < 0.001
*S* = 1.07	Δρ_max_ = 0.12 e Å^−^^3^
529 reflections	Δρ_min_ = −0.15 e Å^−^^3^
69 parameters	Extinction correction: SHELXL97 (Sheldrick, 2008), Fc^*^=kFc[1+0.001xFc^2^λ^3^/sin(2θ)]^-1/4^
Primary atom site location: structure-invariant direct methods	Extinction coefficient: 0.013 (2)
Secondary atom site location: difference Fourier map	

### Special details

**Table:**

Geometry. All e.s.d.'s (except the e.s.d. in the dihedral angle between two l.s. planes) are estimated using the full covariance matrix. The cell e.s.d.'s are taken into account individually in the estimation of e.s.d.'s in distances, angles and torsion angles; correlations between e.s.d.'s in cell parameters are only used when they are defined by crystal symmetry. An approximate (isotropic) treatment of cell e.s.d.'s is used for estimating e.s.d.'s involving l.s. planes.
Refinement. Refinement of *F*^2^ against ALL reflections. The weighted *R*-factor *wR* and goodness of fit *S* are based on *F*^2^, conventional *R*-factors *R* are based on *F*, with *F* set to zero for negative *F*^2^. The threshold expression of *F*^2^ > σ(*F*^2^) is used only for calculating *R*-factors(gt) *etc*. and is not relevant to the choice of reflections for refinement. *R*-factors based on *F*^2^ are statistically about twice as large as those based on *F*, and *R*- factors based on ALL data will be even larger.

### Fractional atomic coordinates and isotropic or equivalent isotropic displacement parameters (Å^2^)

**Table:**

	*x*	*y*	*z*	*U*_iso_*/*U*_eq_	
N1	0.2116 (4)	0.41727 (11)	0.15388 (16)	0.0350 (5)	
C1	0.1130 (6)	0.48556 (11)	0.1948 (2)	0.0364 (6)	
O1	0.4527 (4)	0.41624 (10)	0.06569 (15)	0.0456 (5)	
H1	0.357 (7)	0.4338 (15)	−0.012 (3)	0.072 (10)*	
C2	0.1068 (5)	0.34702 (11)	0.2004 (2)	0.0332 (5)	
O2	0.1965 (5)	0.54622 (9)	0.14878 (15)	0.0531 (6)	
C3	0.2131 (7)	0.27901 (13)	0.1487 (2)	0.0456 (7)	
H3	0.3541	0.2788	0.0808	0.055*	
C4	0.1046 (7)	0.21177 (13)	0.2002 (2)	0.0557 (8)	
H4	0.1745	0.1659	0.1669	0.067*	

### Atomic displacement parameters (Å^2^)

**Table:**

	*U*^11^	*U*^22^	*U*^33^	*U*^12^	*U*^13^	*U*^23^
N1	0.0369 (10)	0.0401 (9)	0.0279 (8)	0.0022 (10)	0.0011 (9)	−0.0011 (8)
C1	0.0452 (15)	0.0371 (12)	0.0269 (10)	−0.0036 (11)	−0.0014 (13)	−0.0019 (9)
O1	0.0416 (9)	0.0631 (10)	0.0320 (8)	0.0113 (10)	0.0045 (9)	0.0052 (9)
C2	0.0354 (14)	0.0334 (10)	0.0309 (10)	0.0004 (10)	−0.0096 (12)	−0.0007 (8)
O2	0.0823 (16)	0.0374 (8)	0.0397 (9)	−0.0123 (10)	0.0136 (13)	0.0017 (7)
C3	0.0519 (16)	0.0435 (13)	0.0415 (13)	0.0099 (13)	−0.0118 (16)	−0.0077 (10)
C4	0.072 (2)	0.0336 (11)	0.0615 (16)	0.0086 (13)	−0.0242 (18)	−0.0079 (11)

### Geometric parameters (Å, °)

**Table:**

N1—C1	1.345 (3)	C2—C2^i^	1.381 (4)
N1—O1	1.381 (2)	C2—C3	1.391 (3)
N1—C2	1.404 (3)	C3—C4	1.382 (3)
C1—O2	1.226 (3)	C3—H3	0.9300
C1—C1^i^	1.503 (4)	C4—C4^i^	1.372 (6)
O1—H1	0.96 (3)	C4—H4	0.9300
			
C1—N1—O1	117.21 (19)	C2^i^—C2—N1	118.08 (11)
C1—N1—C2	125.42 (18)	C3—C2—N1	121.5 (2)
O1—N1—C2	117.18 (18)	C4—C3—C2	118.6 (2)
O2—C1—N1	124.4 (2)	C4—C3—H3	120.7
O2—C1—C1^i^	119.22 (14)	C2—C3—H3	120.7
N1—C1—C1^i^	116.41 (12)	C4^i^—C4—C3	120.93 (16)
N1—O1—H1	104.2 (17)	C4^i^—C4—H4	119.5
C2^i^—C2—C3	120.47 (15)	C3—C4—H4	119.5
			
O1—N1—C1—O2	8.8 (3)	C1—N1—C2—C3	177.4 (2)
C2—N1—C1—O2	−176.4 (2)	O1—N1—C2—C3	−7.8 (3)
O1—N1—C1—C1^i^	−170.8 (2)	C2^i^—C2—C3—C4	−1.1 (4)
C2—N1—C1—C1^i^	4.1 (4)	N1—C2—C3—C4	178.9 (2)
C1—N1—C2—C2^i^	−2.5 (4)	C2—C3—C4—C4^i^	0.3 (5)
O1—N1—C2—C2^i^	172.3 (2)		

Symmetry codes: (i) −*x*, *y*, −*z*+1/2.

### Hydrogen-bond geometry (Å, °)

**Table:**

*D*—H···*A*	*D*—H	H···*A*	*D*···*A*	*D*—H···*A*
O1—H1···O2^ii^	0.96 (3)	1.63 (3)	2.584 (2)	174 (3)

Symmetry codes: (ii) *x*, −*y*+1, −*z*.
